# Efficacy of glucocorticoids, vitamin A and caffeine therapies for neonatal mortality in preterm infants: a network meta-analysis

**DOI:** 10.18632/oncotarget.20882

**Published:** 2017-09-14

**Authors:** Ying Li, Jie Gao, Qiwei Wang, Xiaojian Ma

**Affiliations:** ^1^ Department of Paediatrics, Huaihe Hospital, Henan University, Kaifeng 475000, Henan, China

**Keywords:** neonatal mortality, efficacy, treatments, network meta-analysis

## Abstract

**Introduction:**

The paper aimed to evaluate the efficacy of different therapies in improving survival among preterm infants.

**Materials and Methods:**

PubMed and Embase were searched from inception to 2017. We assessed studies for eligibility and extracted data. A Bayesian random-effects model was used to evaluate different therapies combined direct comparisons with indirect evidence. Consistency analysis was achieved using node-splitting plots. Surface under the cumulative ranking curve (SUCRA) was calculated to rank different therapies. Rankings of the competing therapies were also performed.

**Results:**

A total of 42 randomized controlled trials (RCTs) were included for the network meta-analysis. Forest plots demonstrated that dexamethasone (OR = 10.13, 95% CrI: 5.11 to 17.89) and vitamin A (OR = 28.44, 95% CrI: 14.66 to 42.11) is superior to placebo in duration of oxygen supplementation while vitamin A (OR = −29.76, 95% CrI: −57.66 to −1.75) is inferior to placebo with regard to duration of hospitalization. Also, dexamethasone (OR = 0.42, 95% CrI: 0.24 to 0.68) showed lower incidence rate of BPD.

SUCRA results showed the superiority of Budesonide based on primary efficacy outcomes. In addition, dexamethasone also showed high efficacy ranking in duration of ventilation, duration of oxygen supplementation, and occurrence of BPD. Hydrocortisone was effective in reducing neonatal mortality. No significant difference was found among these drugs.

**Conclusions:**

No significant heterogeneity was found among these drugs. In general, budesonide might have the potential to be the optimal drug for its efficacy in reducing neonatal mortality and BPD, the two most essential outcome measures. Dexamethasone might be the suboptimal drug.

## INTRODUCTION

In resource poor countries it is important to use scares resources on the treatments that are most likely to have the greatest benefit. Worldwide neonatal mortality and morbidity are too high, according to the statistical research, 2.9 million newborn babies died within 28 days in 2012 [1]. The perinatal mortality in developing countries like Pakistan was four times higher than in developed countries [2].

There are three main causes accounting for most of the neonatal mortalities: preterm birth, bronchopulmonary dysplasia (BPD), and birth defects. The chance of survival in preterm varied from 80% with 25 weeks, 55% with 24 weeks, to 15% with 23 weeks [3]. As for other two factors, BPD could cause ventilator failure among newborn infants and even apnea [4]. Besides, birth defects associated with an increased risk of infant mortality, accounting for approximately 20% of neonatal mortalities [5].

Many drugs were found to have potential effect to reduce preterm mortality such as glucocorticoids, vitamin A, and caffeine [6–10]. Glucocorticoids, including beclomethasone, budesonide, dexamethasone, fluticasone, and hydrocortisone, might also take part in the feedback mechanism of the immune system pharmacologically to treat immune diseases [11]. Dexamethasone was a systemic corticosteroid that could decrease the occurrence of BPD and reduce the period of ventilation while increasing the potential risk of hypertension, infections and hyperglycemia [10]. Hydrocortisone, a systemic corticosteroid, was used to improve survival rate among preterm babies without BPD [10]. However, previous researches suggested that systemic corticosteroids not only performed well in efficacy but also had more serious side effects. Thus, beclomethasone, a commonly used inhaled corticosteroid like fluticasone [8], was utilized to substitute systemic corticosteroids [12] and budesonide was an aerosolized corticosteroid with reduced side effects [13]. In addition to glucocorticoids, vitamin A, as another potential drug, was important in building up the ductus arteriosus and bronchopulmonary function [14]. Besides, caffeine was used to prevent neonatal babies from apnea [7].

There have been numerous meta-analyses (MA) relevant to our study in the last two decades. Yet there existed several deficiencies in these meta-analyses. First of all, a great majority maintained that RCTs and patients were inadequate in quantity, leading to an unsatisfying sample size [15, 16]. Also, a large portion of previous MA implied that a lack of long-term follow-up of infants confined the clarity of drug efficacy and adverse effect [17–21]. Furthermore, many indicated that open-label prescriptions contaminated study arms thus made it harder to evaluate the effect of a certain therapy alone [22–24]. It should not be ignored that some MA claimed evidence of doubtful clinical relevance [25, 26]. Besides, some recent studies failed to process the heterogeneity of data [25, 27].

Due to all the inadequacies of existent MA mentioned above, and the fact that network meta-analysis (NMA) combines both direct and indirect evidence, we consider NMA as an appropriate strategy both to cover the shortage of MA and to increase statistical power. Therefore, we conduct this NMA in order to assess the efficacy of eight therapies in improving survival among preterm infants. We chose neonatal mortality and incidence of BPD as primary efficacy outcomes. Duration of ventilation, duration of oxygen supplementation, and duration of hospital stay are secondary outcomes in this NMA.

## RESULTS

### Literature search

**Figure 1:**
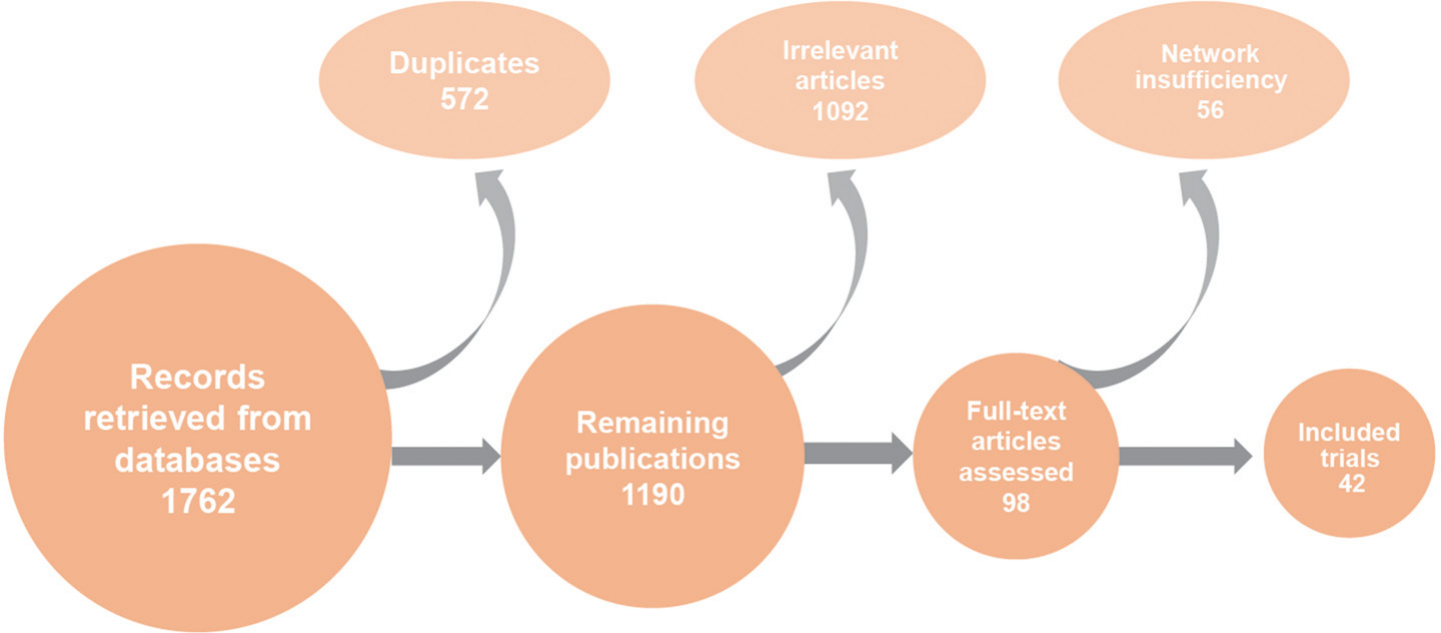
Flow chart of publication screening process

**Figure 2:**
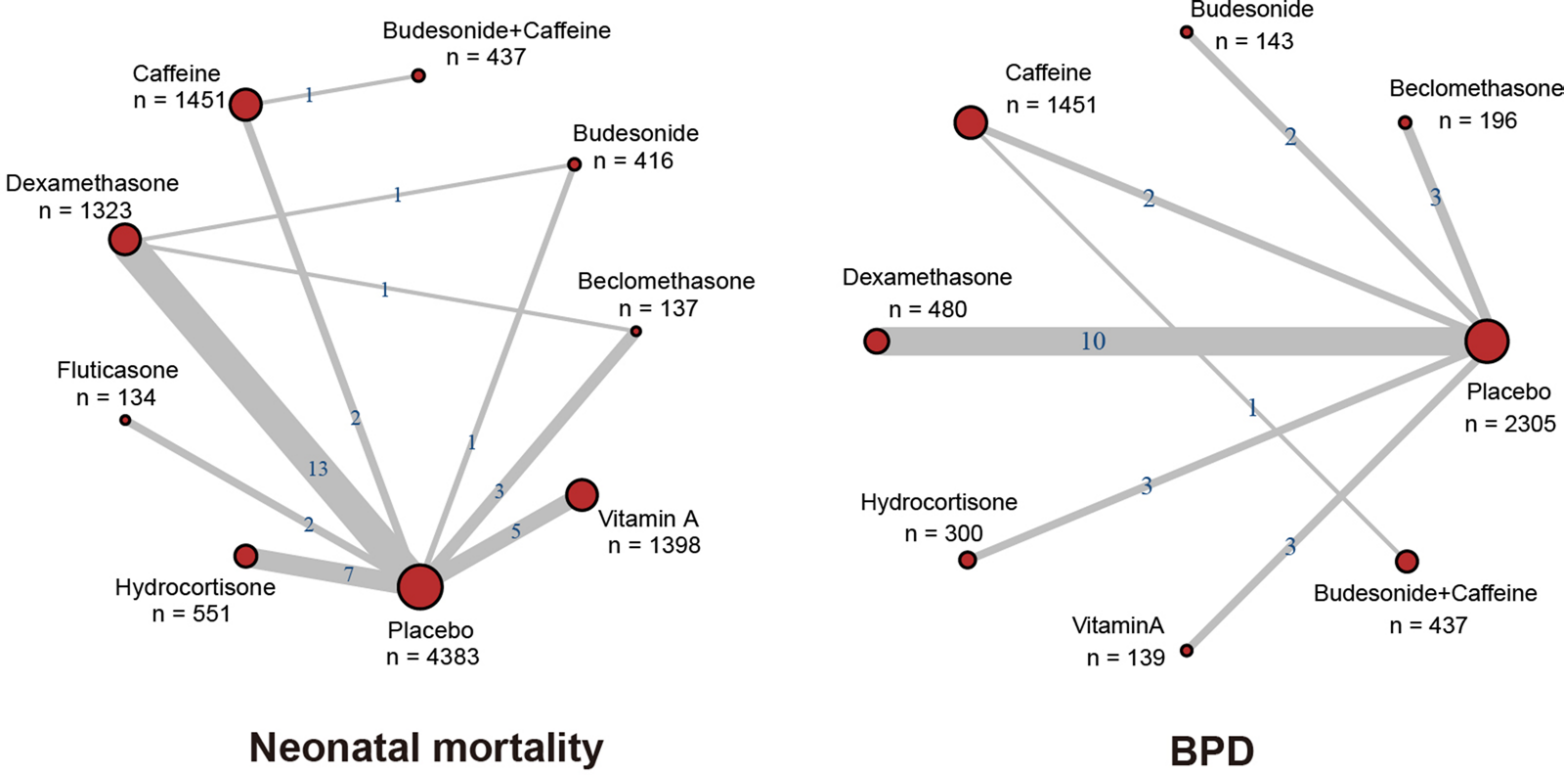
Network diagram Each node represents a therapy; the number beside the nodes represents the number of people involved and the number between two nodes represents the number of study involved in the head-to-head comparison.

As shown in Figure 1, we identified 1762 records after an initial screening in this NMA through electronic database searching. Then we removed 572 records identified as duplicates. Only 98 among 1190 remaining studies were relevant and hence assessed. 56 of those relevant articles were excluded after a review of full texts. Eventually 42 RCTs were included in this study through quantitative synthesis (Supplementary Table 1) [4, 6–10, 12–14, 28–60]. The publication year of these included papers ranged from 1995 to 2017. The majority of them were published before 2006. Moreover, 36 out of 42 trials included neonatal mortality, 21 trials included duration of ventilation, 17 trials included duration of hospital stay, 9 trials included the occurrence of BPD, and 19 trials considered duration of oxygen supplementation. The network plots were shown in Figure 2 and Supplementary Figure 1.

### Primary outcomes

**Figure 3:**
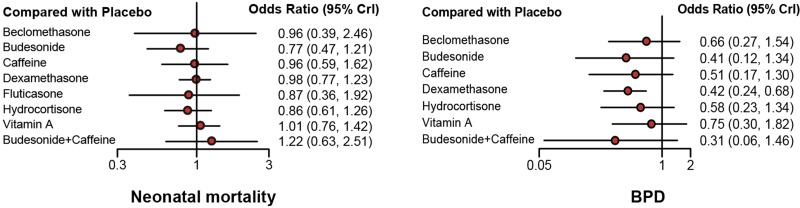
Forest plots of all outcomes Odds ratio (ORs) or mean difference (MD) with corresponding 95% credible intervals (95% CrIs) was used to measure the relative efficacy of different treatments.

As was shown in Table 1 and Figure 3, a total of 42 trials including 10625 infants reported the neonatal mortality and BPD as primary outcomes. No statistical difference was observed with respect to neonatal mortality. Dexamethasone showed lower incidence rate of BPD (OR = 0.42, 95% CrI: 0.24 to 0.68) than placebo.

**Table 1:** Results of primary and secondary outcomes

**Neonatal** **mortality**	Placebo	5.77 (−6.32, 17.65)	1.57 (−10.08, 13.28)	2.01 (−13.98, 17.89)	4.24 (−2.69, 11.55)	−1.9 (−18.12, 14.13)	4.01 (−2.82, 10.73)	8.58 (−3.03, 19.94)	2.31 (−20.14, 24.64)	**Duration of** **ventilation**
	0.96 (0.39, 2.46)	Beclomethasone	−4.16 (−20.74, 12.58)	−3.74 (−23.57, 16.22)	−1.53 (−15.22, 12.65)	−7.74 (−27.53, 12.5)	−1.75 (−15.54, 12)	2.82 (−13.93, 19.39)	−3.47 (−28.51, 21.84)	
	0.77 (0.47, 1.21)	0.79 (0.28, 2.14)	Budesonide	0.44 (−19.28, 20.07)	2.62 (−10.92, 16.51)	−3.52 (−23.42, 16.3)	2.41 (−11.18, 15.8)	6.99 (−9.55, 23.26)	0.66 (−24.66, 25.86)	
	0.96 (0.59, 1.62)	1.01 (0.35, 2.89)	1.25 (0.65, 2.56)	Caffeine	2.21 (−14.93, 19.8)	−3.92 (−26.6, 18.9)	2.01 (−15.42, 19.35)	6.59 (−13.24, 26.04)	0.29 (−15.57, 15.97)	
	0.98 (0.77, 1.23)	1.03 (0.39, 2.53)	1.27 (0.83, 1.97)	1.02 (0.57, 1.73)	Dexamethasone	−6.13 (−23.93, 11.22)	−0.21 (−10.34, 9.35)	4.38 (−9.59, 17.49)	−1.91 (−25.8, 21.11)	
	0.87 (0.36, 1.92)	0.91 (0.23, 3.16)	1.14 (0.45, 2.89)	0.91 (0.34, 2.27)	0.89 (0.36, 2.05)	Fluticasone	5.92 (−11.76, 23.39)	10.5 (−9.47, 30.21)	4.24 (−23.48, 31.65)	
	0.86 (0.61, 1.26)	0.89 (0.34, 2.41)	1.12 (0.64, 2.05)	0.90 (0.49, 1.65)	0.88 (0.59, 1.38)	0.98 (0.42, 2.53)	Hydrocortisone	4.58 (−8.83, 17.82)	−1.67 (−25.04, 21.78)	
	1.01 (0.76, 1.42)	1.07 (0.41, 2.77)	1.36 (0.79, 2.41)	1.09 (0.59, 1.97)	1.06 (0.73, 1.58)	1.21 (0.51, 3.12)	1.21 (0.74, 1.95)	Vitamin A	−6.34 (−31.47, 18.98)	
	1.22 (0.63, 2.51)	1.30 (0.41, 4.18)	1.63 (0.72, 3.82)	1.32 (0.79, 2.11)	1.26 (0.63, 2.72)	1.42 (0.51, 4.31)	1.43 (0.66, 3.10)	1.19 (0.55, 2.59)	Budesonide+Caffeine	
**BPD**	Placebo	1.18 (−11, 11.79)	3.88 (−8.39, 18.79)	8.26 (−8.94, 25.22)	10.13 (5.11, 17.89)	0.81 (−5.14, 10.5)	28.44 (14.66, 42.11)	9.74 (−11.91, 30.91)	**Duration of** **oxygen supplement**	
	0.66 (0.27, 1.54)	Beclomethasone	2.77 (−12.95, 22.51)	7.07 (−12.76, 28.31)	9.05 (−2.49, 24.4)	−0.1 (−11.96, 16.23)	27.09 (10.71, 46.03)	8.53 (−14.68, 33.66)		
	0.41 (0.12, 1.34)	0.61 (0.14, 2.75)	Budesonide	4.25 (−18.74, 24.78)	6.17 (−6.58, 19.02)	−2.85 (−18.15, 12.76)	24.61 (3.92, 42.36)	5.79 (−21.02, 29.74)		
	0.51 (0.17, 1.30)	0.76 (0.19, 2.77)	1.25 (0.24, 5.64)	Caffeine	1.94 (−15.18, 21.44)	−7.19 (−24.45, 13.07)	20.14 (−1.44, 42.12)	1.48 (−11.88, 14.76)		
	0.42 (0.24, 0.68)	0.64 (0.23, 1.71)	1.05 (0.27, 3.67)	0.84 (0.28, 2.66)	Dexamethasone	−8.92 (−18.85, 0.96)	18.58 (1.73, 31.71)	−0.34 (−24.24, 20.55)		
	0.58 (0.23, 1.34)	0.87 (0.25, 2.97)	1.43 (0.31, 6.05)	1.14 (0.31, 4.53)	1.36 (0.49, 3.78)	Hydrocortisone	27.61 (9.81, 41.36)	8.73 (−15.87, 29.73)		
	0.75 (0.30, 1.82)	1.13 (0.32, 3.97)	1.86 (0.40, 8.17)	1.49 (0.41, 6.05)	1.77 (0.64, 5.16)	1.30 (0.37, 4.71)	Vitamin A	−18.71 (−44.12, 6.6)		
	0.31 (0.06, 1.46)	0.47 (0.07, 2.77)	0.76 (0.09, 5.37)	0.61 (0.17, 2.16)	0.73 (0.13, 3.94)	0.53 (0.08, 3.29)	0.41 (0.06, 2.53)	Budesonide+Caffeine		
	Placebo									
**Duration of** **hospital stay**	6.17 (−5.77, 17.82)	Beclomethasone								
	−5.41 (−23.25, 12.43)	−11.53 (−32.68, 10.04)	Caffeine							
	2.59 (−7.33, 13.28)	−3.58 (−18.09, 12.41)	7.88 (−12.25, 29.24)	Dexamethasone						
	5.65 (−1.06, 12.6)	−0.48 (−13.85, 13.46)	11.04 (−7.92, 30.42)	3.16 (−9.66, 14.94)	Hydrocortisone					
	26.41 (12.83, 39.82)	20.23 (2.76, 38.41)	31.83 (9.4, 54)	23.95 (6.12, 40.07)	20.77 (5.29, 35.78)	Vitamin A				
	−3.4 (−27.72, 21.46)	−9.4 (−36.49, 17.69)	1.99 (−14.8, 18.93)	−5.77 (−32.99, 19.87)	−9.03 (−34.47, 16.44)	−29.76 (−57.66, −1.75)	Budesonide+Caffeine			

Note: Treatments in the row are compared to the treatments in the column. Results of mortality and BPD are expressed as odd ratio with 95% credible intervals (CrIs), duration of ventilation, oxygen supplement and hospital stay are expressed with mean difference with 95% CrIs. Bold numbers indicate significant results (*p* < 0.05). BPD: bronchopulmonary dysplasia

### Secondary outcomes

No statistical difference was observed regarding the duration of ventilation according to Table 1 and Supplementary Figure 2, vitamin A appeared to be optimal concerning the duration of hospital stay (MD = −29.76, 95% CrI: −57.66 to −1.75) and oxygen supplement (MD = 28.44, 95% CrI: 14.66 to 42.11) compared with placebo. Dexamethasone is also superior to placebo in duration of oxygen supplementation (MD = 10.13, 95% CrI: 5.11 to 17.89).

### Ranking probabilities from SUCRA

SUCRA was performed (Table 2) to examine the relative performance of different therapies. Budesonide ranked the first in neonatal mortality (SUCRA: 0.766) and showed satisfying efficacy in reducing the occurrence of BPD (SUCRA: 0.678) but the performance was moderate in duration of oxygen supplementation (SUCRA: 0.425) and duration of ventilation (SUCRA: 0.407). Vitamin A ranked first in terms of all secondary outcomes, including duration of ventilation (SUCRA: 0.780), oxygen supplement (SUCRA: 0.982), and hospital stay (SUCRA: 0.990). Budesonide combined with caffeine was the most ideal therapy in reducing the occurrence of BPD (SUCRA: 0.789) and ranked third in duration of oxygen (SUCRA: 0.622), yet performed poor at lowering neonatal mortality (SUCRA: 0.232). Hydrocortisone proved to be suboptimal in both neonatal mortality (SUCRA: 0.658) and duration of hospital stay (SUCRA: 0.634). Beclomethasone ranked second in duration of ventilation (SUCRA: 0.645) and finished third in duration of hospital stay (SUCRA: 0.623). Dexamethasone showed high efficacy in duration of ventilation (SUCRA: 0.585) and duration of oxygen supplementation (SUCRA: 0.700), but its performance regarding neonatal mortality (SUCRA: 0.447) was not desirable.

**Table 2:** Surface under the cumulative ranking curves (SUCRA) for each outcome

Drugs	Neonatal mortality	BPD	Duration of ventilation	Duration of oxygen supplement	Duration of hospital stay
Beclomethasone	0.495	0.389	**0.645**	0.279	**0.623**
Budesonide	**0.766**	**0.678**	0.425	0.407	-
Caffeine	0.523	0.553	0.454	0.550	0.185
Dexamethasone	0.447	**0.697**	**0.585**	**0.700**	0.464
Fluticasone	**0.587**	-	0.285	-	-
Hydrocortisone	**0.658**	0.481	0.571	0.268	**0.634**
Placebo	0.422	0.097	0.280	0.192	0.307
Vitamin A	0.370	0.316	**0.780**	**0.982**	**0.990**
Budesonide+Caffeine	0.232	**0.789**	0.476	**0.622**	0.296

Note: A higher value suggests a higher probability of ranking first in related outcomes. BPD: bronchopulmonary dysplasia.

### Consistency analysis

The results of consistency analysis were exhibited in Table 3. As was shown in the node-splitting plots, no inconsistency between direct and indirect evidence was observed as all the *P* values were larger than 0.05.

**Table 3:** Node-splitting result

Treatment			Comparison			*p*-value
			direct	indirect	network	
**Neonatal mortality**						
Beclomethasone	vs.	Placebo	1.30 (0.39, 3.70)	0.37 (0.06, 2.31)	0.96 (0.37, 2.52)	0.245
Budesonide	vs.	Placebo	0.75 (0.36, 1.62)	0.78 (0.42, 1.41)	0.76 (0.47, 1.22)	0.918
Dexamethasone	vs.	Placebo	0.97 (0.76, 1.33)	1.21 (0.52, 2.90)	0.98 (0.76, 1.21)	0.603
Dexamethasone	vs.	Beclomethasone	2.51 (0.39, 15.0)	0.68 (0.20, 2.41)	1.01 (0.38, 2.51)	0.265
Dexamethasone	vs.	Budesonide	1.31 (0.72, 2.25)	1.32 (0.59, 3.02)	1.31 (0.85, 2.01)	0.985
**Duration of oxygen supplement**						
Budesonide	vs.	Placebo	−7.12 (−36.2, 19.1)	−3.44 (−23.8, 13.2)	−4.06 (−19.5, 8.32)	0.808
Dexamethasone	vs.	Placebo	−10.2 (−18.1, −5.2)	−13.2 (−45.1, 16.2)	−10.3 (−19.2, −5.3)	0.834
Dexamethasone	vs.	Budesonide	−6.72 (−22.7, 7.92)	−3.42 (−32.2, 23.7)	−6.17 (−20.1, 5.54)	0.816
**Duration of hospital stay**						
Beclomethasone	vs.	Placebo	−6.91 (−19.3, 6.02)	6.77 (−43.1, 53.2)	−6.02 (−17.5, 6.77)	0.611
Dexamethasone	vs.	Placebo	−2.23 (−13.4, 8.01)	−15.5 (−65.2, 36.6)	−2.24 (−13.3, 7.56)	0.630
Dexamethasone	vs.	Beclomethasone	−6.57 (−55.7, 42.2)	4.87 (−14.5, 20.4)	3.72 (−12.7, 18.01)	0.678

Note: Results of mortality are expressed as odd ratio with 95% credible intervals (CrIs), duration of oxygen supplement and hospital stay are expressed with mean difference with 95% CrIs to determine the difference between direct and indirect evidence.

## DISCUSSION

This NMA aimed to evaluate the performance of seven single therapies and one combination drug in reducing neonatal mortality and the occurrence of BPD among preterm infants. In addition, secondary outcomes including duration of ventilation, duration of oxygen, and duration of hospital stay were also evaluated to identify the efficacy of different drugs. Note that duration of hospital stay might be affected by multiple variables, making comparison cross studies difficult.

According to our results, budesonide was associated with a tendency towards a lower mortality rate and incidence of BPD compared with other therapies. Yet its performance concerning duration of ventilation and duration of oxygen supplement is relatively undesirable. Also, delivery methods of budesonide might influence its drug effects to a great extent, which is not analyzed in our study. For example, inhaled budesonide therapy for BPD decreases the need for mechanical ventilation similar to intravenous dexamethasone, whereas basically did not trigger significant side effects [13]. Therefore further studies on delivery methods of drugs are compelling.

Also we demonstrated that vitamin A ranked first with respect to duration of ventilation, duration of oxygen, and duration of hospital stay, which was consistent with existing studies [37, 51]. Yet there was no significant trend towards the reduction of mortality and BPD among preterm infants [6, 14, 29, 37, 51]. Thus, further researches on both budesonide and vitamin A are necessary to see whether outcomes turn out to be satisfactory and balanced without adverse side effects.

Dexamethasone ranked second with respect to mechanical oxygen supply and rated third place in ventilation whereas exhibited little effect on reducing neonatal mortalities. As demonstrated in a study designed by Doyle et al., low-dose dexamethasone did shorten the duration of intubation among ventilator-dependent infants [9]. Also, according to Walther et al., there was a trend for fewer days on the ventilator and on oxygen in the dexamethasone group compared to the control group [53]. The result of another study carried out by Anttila et al. showed that the early prolonged dexamethasone therapy had a significant beneficial effect by reducing the incidence of BPD, but no significant effect on mortality [24]. Also whether the advantages of dexamethasone as a therapy in BPD overbalance its adverse effects or not remains unclear [21]. Thus long-term follow-ups and reports of late outcomes are in urgent needs.

When it comes to combination therapy, budesonide and caffeine co-intervention had the potential to be the most effective drug in reducing BPD among preterm infants, yet this combination might concurrently increase mortality rate. Moreover, the short-term outcomes were not completely valid for the treatment of neonatal mortality in preterm infants, and hence an overall assessment of the efficacy and associated risks of inhaled budesonide were needed [28]. Besides, considering only one trial concerning co-intervention was included in our study [28] and many of the therapies that were considered are used simultaneously in clinical practice, there is a need for large RCTs to explore the effects of a certain therapy in combination with various types of therapies. For example, our results imply that vitamin A and budesonide should be used jointly in future trials to examine whether these two drugs could complement each other.

### Limitations

Although conducted as meticulously as possible, however, this study still has some limitations.

Firstly, an imbalance of a great degree in terms of the quality of evidence for each therapy could decrease the power and reliability of the results. For example, under the outcome of mortality, the sample size of beclomethasone was much smaller than that of dexamethasone; some treatments were even not investigated under some outcomes; researches on co-intervention were not sufficient, which resulted into lack of powerful references. Moreover, outcomes concerning safety were not included in this study due to the lack of evidence, which was of equal importance when it comes to clinical practice. Thus, more efforts remained to be made to reach a more comprehensive conclusion.

Secondly, study designs such as methods for application and dosing schedules might also influence the efficacy of therapies. For example, budesonide could be taken by systematic or inhaled [35]. Yet application methods and dosing schedules were not examined in our analysis due to the limited amount of relevant studies. The effect of study design on outcomes remained unknown in our study.

Besides, previous study suggested that some characteristics of infants such as birth weight and lung developmental stages were vital when measuring the neonatal mortality [61]. However, in this study, patients characteristics were not taken into consideration and as it differed from trials to trials thus raising the question of study bias and validity. Hence, more comprehensive large-sample studies covering more outcomes should be conducted to reach a more grounded conclusion also to serve as more reliable clinical references.

## CONCLUSIONS

Budesonide might have the potential to be the optimal drug for its efficacy in reducing neonatal mortality and the incidence of BPD. Dexamethasone could be suboptimal for its high ranking in three outcome measures. However, there is no significant difference observed among these drugs in terms of neonatal mortality. Moreover, there is an urgent need for studies concerning co-interventions considering current situation of clinical practice.

## MATERIALS AND METHODS

We conducted our NMA in accordance with the methods recommended by the Preferred Reporting Items for Systematic Reviews and Meta-Analyses guidelines.

### Search strategy and selection criteria

PubMed and Embase were searched from inception until 2017. We evaluated preterm infants with five outcomes including neonatal mortality, BPD, duration of ventilation, duration of oxygen, duration of hospitalization, and seven therapies including beclomethasone, budesonide, dexamethasone, fluticasone, hydrocortisone, vitamin A, and caffeine. The five outcomes, seven therapies and “randomized controlled trials” were identified as main keywords with their corresponding synonyms combined.

Studies should meet all the following criteria to be eligible for this NMA: (1) studies were designed to be randomized controlled trials (RCTs); (2) studies should contain at least one pair of comparison among the included therapies; (3) the outcomes should at least one of the investigated outcomes. Duplicates and studies that failed to meet the criteria were excluded.

### Data extraction

Data extraction was processed by two reviewers independently. The following data were extracted from original studies: (1) publication year, author, and country; (2) basic information of the newborn, including sample size, gestational age, birth weight, and characteristics of subjects; (3) drugs and methods used in study groups and control groups; (4) neonatal mortality, BPD, duration of ventilation, duration of oxygen supplementation, and duration of hospital stay. Duration of oxygen supplementation was defined as the time in days from neonatal intensive care unit arrival to the time oxygen was permanently discontinued [62]; duration of ventilation means the length (days) infants treated with ventilation therapy; and hospital stay duration means the length (days) infants stayed in the hospital.

### Statistical analysis

Network meta-analyses yield direct pair-wise effects estimations (e.g., *A* vs. *B*) and indirect effects estimations (e.g., *A* vs. *B* via *C* using comparisons of both *A* vs. *C* and *C* vs. *B*). Accordingly, NMA is able to valuate network effects combining direct and indirect effects and rank the interventions thus allow the selection of the best among multiple therapies. The relative efficacy between several therapies is calculated no matter if they were directly compared in the original trials. Our NMA is performed by the aid of R (V 3.3.2) and STATA (V 13.0). Odds ratios (ORs) with 95% credible intervals (CrIs) were employed to measure neonatal mortality and BPD. The CrIs were calculated by a Bayesian random-effects model. Moreover, duration of ventilation, duration of oxygen supplementation, and duration of hospital stay were evaluated by mean difference (MD) with 95% CrIs. As a summary index ranging from 0 to 1, surface under the cumulative ranking curve (SUCRA) signifies to what degree a therapy is better or worse than other therapies. A higher SUCRA value indicates a better performance. SUCRA was calculated in this NMA to show the ranking of different therapies probabilities that a therapy is above a certain ranking under different outcomes. Node-splitting plots were computed to analyze the consistency between direct and indirect comparisons. Besides, a Jaded scale score table was made to examine the quality of included trials (Supplementary Table 2).

## SUPPLEMENTARY MATERIALS FIGURES AND TABLE





## References

[1] Turab A, Pell LG, Bassani DG, Soofi S, Ariff S, Bhutta ZA, Morris SK (2014). The community-based delivery of an innovative neonatal kit to save newborn lives in rural Pakistan: design of a cluster randomized trial. BMC Pregnancy Childbirth.

[2] Norton M (2005). New evidence on birth spacing: promising findings for improving newborn, infant, child, and maternal health. Int J Gynaecol Obstet.

[3] Frey HA, Klebanoff MA (2016). The epidemiology, etiology, and costs of preterm birth. Semin Fetal Neonatal Med.

[4] Armanian AM, Iranpour R, Faghihian E, Salehimehr N (2016). Caffeine Administration to Prevent Apnea in Very Premature Infants. Pediatr Neonatol.

[5] Almli LM, Alter CC, Russell RB, Tinker SC, Howards PP, Cragan J, Petersen E, Carrino GE, Reefhuis J (2017). Association Between Infant Mortality Attributable to Birth Defects and Payment Source for Delivery - United States, 2011–2013. MMWR Morb Mortal Wkly Rep..

[6] Wardle SP, Hughes A, Chen S, Shaw NJ (2001). Randomised controlled trial of oral vitamin A supplementation in preterm infants to prevent chronic lung disease. Arch Dis Child Fetal Neonatal Ed.

[7] Schmidt B, Roberts RS, Davis P, Doyle LW, Barrington KJ, Ohlsson A, Solimano A, Tin W, Caffeine for Apnea of Prematurity Trial Group (2006). Caffeine therapy for apnea of prematurity. N Engl J Med.

[8] Fok TF, Lam K, Dolovich M, Ng PC, Wong W, Cheung KL, So KW (1999). Randomised controlled study of early use of inhaled corticosteroid in preterm infants with respiratory distress syndrome. Arch Dis Child Fetal Neonatal Ed.

[9] Doyle LW, Davis PG, Morley CJ, McPhee A, Carlin JB (2006). Low-dose dexamethasone facilitates extubation among chronically ventilator-dependent infants: a multicenter, international, randomized, controlled trial. Pediatrics.

[10] Baud O, Maury L, Lebail F, Ramful D, El Moussawi F, Nicaise C, Zupan-Simunek V, Coursol A, Beuchee A, Bolot P, Andrini P, Mohamed D, Alberti C (2016). Effect of early low-dose hydrocortisone on survival without bronchopulmonary dysplasia in extremely preterm infants (PREMILOC): a double-blind, placebo-controlled, multicentre, randomised trial. Lancet.

[11] Baid SK, Nieman LK (2006). Therapeutic doses of glucocorticoids: implications for oral medicine. Oral Diseases.

[12] Cole CH, Colton T, Shah BL, Abbasi S, MacKinnon BL, Demissie S, Frantz ID (1999). Early inhaled glucocorticoid therapy to prevent bronchopulmonary dysplasia. N Engl J Med.

[13] Jonsson B, Eriksson M, Soder O, Broberger U, Lagercrantz H (2000). Budesonide delivered by dosimetric jet nebulization to preterm very low birthweight infants at high risk for development of chronic lung disease. Acta Paediatr.

[14] Ravishankar C, Nafday S, Green RS, Kamenir S, Lorber R, Stacewicz-Sapuntzakis M, Bridges ND, Holzman IR, Gelb BD (2003). A trial of vitamin A therapy to facilitate ductal closure in premature infants. J Pediatr.

[15] Darlow BA, Graham PJ, Rojas-Reyes MX (2016). Vitamin A supplementation to prevent mortality and short- and long-term morbidity in very low birth weight infants. Cochrane Database Syst Rev.

[16] Park HW, Lim G, Chung SH, Chung S, Kim KS, Kim SN (2015). Early Caffeine Use in Very Low Birth Weight Infants and Neonatal Outcomes: A Systematic Review and Meta-Analysis. J Korean Med Sci.

[17] Doyle LW, Ehrenkranz RA, Halliday HL (2014). Early (< 8 days) postnatal corticosteroids for preventing chronic lung disease in preterm infants. Cochrane Database Syst Rev.

[18] Shah SS, Ohlsson A, Halliday HL, Shah VS (2012). Inhaled versus systemic corticosteroids for preventing chronic lung disease in ventilated very low birth weight preterm neonates. Cochrane Database Syst Rev.

[19] Halliday HL, Ehrenkranz RA, Doyle LW (2010). Early (< 8 days) postnatal corticosteroids for preventing chronic lung disease in preterm infants. Cochrane Database Syst Rev.

[20] Halliday HL, Ehrenkranz RA, Doyle LW (2003). Early postnatal (< 96 hours) corticosteroids for preventing chronic lung disease in preterm infants. Cochrane Database Syst Rev.

[21] Arias-Camison JM, Lau J, Cole CH, Frantz ID (1999). Meta-analysis of dexamethasone therapy started in the first 15 days of life for prevention of chronic lung disease in premature infants. Pediatr Pulmonol.

[22] Doyle LW, Ehrenkranz RA, Halliday HL (2010). Dexamethasone treatment in the first week of life for preventing bronchopulmonary dysplasia in preterm infants: a systematic review. Neonatology.

[23] Doyle LW, Ehrenkranz RA, Halliday HL (2010). Postnatal hydrocortisone for preventing or treating bronchopulmonary dysplasia in preterm infants: a systematic review. Neonatology.

[24] Anttila E, Peltoniemi O, Haumont D, Herting E, ter Horst H, Heinonen K, Kero P, Nykanen P, Oetomo SB, Hallman M (2005). Early neonatal dexamethasone treatment for prevention of bronchopulmonary dysplasia. Randomised trial and meta-analysis evaluating the duration of dexamethasone therapy. Eur J Pediatr.

[25] Kua KP, Lee SW (2017). Systematic review and meta-analysis of clinical outcomes of early caffeine therapy in preterm neonates. Br J Clin Pharmacol.

[26] Bhuta T, Ohlsson A (1998). Systematic review and meta-analysis of early postnatal dexamethasone for prevention of chronic lung disease. Arch Dis Child Fetal Neonatal Ed.

[27] Onland W, De Jaegere AP, Offringa M, van Kaam A (2017). Systemic corticosteroid regimens for prevention of bronchopulmonary dysplasia in preterm infants. Cochrane Database Syst Rev.

[28] Bassler D, Plavka R, Shinwell ES, Hallman M, Jarreau PH, Carnielli V, Van den Anker JN, Meisner C, Engel C, Schwab M, Halliday HL, Poets CF (2015). Early Inhaled Budesonide for the Prevention of Bronchopulmonary Dysplasia. N Engl J Med.

[29] Benn CS, Fisker AB, Napirna BM, Roth A, Diness BR, Lausch KR, Ravn H, Yazdanbakhsh M, Rodrigues A, Whittle H, Aaby P (2010). Vitamin A supplementation and BCG vaccination at birth in low birthweight neonates: two by two factorial randomised controlled trial. BMJ.

[30] Beresford MW, Primhak R, Subhedar NV, Shaw NJ (2002). Randomised double blind placebo controlled trial of inhaled fluticasone propionate in infants with chronic lung disease. Arch Dis Child Fetal Neonatal Ed.

[31] Bonsante F, Latorre G, Iacobelli S, Forziati V, Laforgia N, Esposito L, Mautone A (2007). Early low-dose hydrocortisone in very preterm infants: a randomized, placebo-controlled trial. Neonatology.

[32] Brozanski BS, Jones JG, Gilmour CH, Balsan MJ, Vazquez RL, Israel BA, Newman B, Mimouni FB, Guthrie RD (1995). Effect of pulse dexamethasone therapy on the incidence and severity of chronic lung disease in the very low birth weight infant. J Pediatr.

[33] Denjean A, Paris-Llado J, Zupan V, Debillon T, Kieffer F, Magny JF, Desfreres L, Llanas B, Guimaraes H, Moriette G, Voyer M, Dehan M, Breart G (1998). Inhaled salbutamol and beclomethasone for preventing broncho-pulmonary dysplasia: a randomised double-blind study. Eur J Pediatr.

[34] Garland JS, Alex CP, Pauly TH, Whitehead VL, Brand J, Winston JF, Samuels DP, McAuliffe TL (1999). A three-day course of dexamethasone therapy to prevent chronic lung disease in ventilated neonates: a randomized trial. Pediatrics.

[35] Halliday HL, Patterson CC, Halahakoon CW (2001). A multicenter, randomized open study of early corticosteroid treatment (OSECT) in preterm infants with respiratory illness: comparison of early and late treatment and of dexamethasone and inhaled budesonide. Pediatrics.

[36] Jangaard KA, Stinson DA, Allen AC, Vincer MJ (2002). Early prophylactic inhaled beclomethasone in infants less than 1250 g for the prevention of chronic lung disease. Paediatr Child Health.

[37] Kiatchoosakun P, Jirapradittha J, Panthongviriyakul MC, Khampitak T, Yongvanit P, Boonsiri P (2014). Vitamin A supplementation for prevention of bronchopulmonary dysplasia in very-low-birth-weight premature Thai infants: a randomized trial. J Med Assoc Thai.

[38] Kothadia JM, O'Shea TM, Roberts D, Auringer ST, Weaver RG, Dillard RG (1999). Randomized placebo-controlled trial of a 42-Day tapering course of dexamethasone to reduce the duration of ventilator dependency in very low birth weight infants. Pediatrics.

[39] Kugelman A, Peniakov M, Zangen S, Shiff Y, Riskin A, Iofe A, Shoris I, Bader D, Arnon S (2017). Inhaled hydrofluoalkane-beclomethasone dipropionate in bronchopulmonary dysplasia. A double-blind, randomized, controlled pilot study. J Perinatol.

[40] Lin YJ, Yeh TF, Hsieh WS, Chi YC, Lin HC, Lin CH (1999). Prevention of chronic lung disease in preterm infants by early postnatal dexamethasone therapy. Pediatr Pulmonol.

[41] Merz U, Kusenbach G, Hausler M, Peschgens T, Hornchen H (1999). Inhaled budesonide in ventilator-dependent preterm infants: a randomized, double-blind pilot study. Biol Neonate.

[42] Nakamura T, Yonemoto N, Nakayama M, Hirano S, Aotani H, Kusuda S, Fujimura M, Tamura M (2016). Early inhaled steroid use in extremely low birthweight infants: a randomised controlled trial. Arch Dis Child Fetal Neonatal Ed.

[43] Parikh NA, Kennedy KA, Lasky RE, McDavid GE, Tyson JE (2013). Pilot randomized trial of hydrocortisone in ventilator-dependent extremely preterm infants: effects on regional brain volumes. J Pediatr.

[44] Peltoniemi O, Kari MA, Heinonen K, Saarela T, Nikolajev K, Andersson S, Voutilainen R, Hallman M (2005). Pretreatment cortisol values may predict responses to hydrocortisone administration for the prevention of bronchopulmonary dysplasia in high-risk infants. J Pediatr.

[45] Rastogi A, Akintorin SM, Bez ML, Morales P, Pildes RS (1996). A controlled trial of dexamethasone to prevent bronchopulmonary dysplasia in surfactant-treated infants. Pediatrics.

[46] Romagnoli C, Zecca E, Vento G, De Carolis MP, Papacci P, Tortorolo G (1999). Early postnatal dexamethasone for the prevention of chronic lung disease in high-risk preterm infants. Intensive Care Med.

[47] Rozycki HJ, Byron PR, Elliott GR, Carroll T, Gutcher GR (2003). Randomized controlled trial of three different doses of aerosol beclomethasone versus systemic dexamethasone to promote extubation in ventilated premature infants. Pediatr Pulmonol.

[48] Stark AR, Carlo WA, Tyson JE, Papile LA, Wright LL, Shankaran S, Donovan EF, Oh W, Bauer CR, Saha S, Poole WK, Stoll BJ (2001). Adverse effects of early dexamethasone in extremely-low-birth-weight infants. National Institute of Child Health and Human Development Neonatal Research Network. N Engl J Med.

[49] Subhedar NV, Ryan SW, Shaw NJ (1997). Open randomised controlled trial of inhaled nitric oxide and early dexamethasone in high risk preterm infants. Arch Dis Child Fetal Neonatal Ed.

[50] Tapia JL, Ramirez R, Cifuentes J, Fabres J, Hubner ME, Bancalari A, Mercado ME, Standen J, Escobar M (1998). The effect of early dexamethasone administration on bronchopulmonary dysplasia in preterm infants with respiratory distress syndrome. J Pediatr.

[51] Tyson JE, Wright LL, Oh W, Kennedy KA, Mele L, Ehrenkranz RA, Stoll BJ, Lemons JA, Stevenson DK, Bauer CR, Korones SB, Fanaroff AA (1999). Vitamin A supplementation for extremely-low-birth-weight infants. National Institute of Child Health and Human Development Neonatal Research Network. N Engl J Med.

[52] Anttila E, Peltoniemi O, Haumont D, Herting E, ter Horst H, Heinonen K, Kero P, Nykanen P, Oetomo SB, Hallman M (2005). Early neonatal dexamethasone treatment for prevention of bronchopulmonary dysplasia. Randomised trial and meta-analysis evaluating the duration of dexamethasone therapy. Eur J Pediatr.

[53] Walther FJ, Findlay RD, Durand M (2003). Adrenal suppression and extubation rate after moderately early low-dose dexamethasone therapy in very preterm infants. Early Hum Dev.

[54] Watterberg KL, Gerdes JS, Cole CH, Aucott SW, Thilo EH, Mammel MC, Couser RJ, Garland JS, Rozycki HJ, Leach CL, Backstrom C, Shaffer ML (2004). Prophylaxis of early adrenal insufficiency to prevent bronchopulmonary dysplasia: a multicenter trial. Pediatrics.

[55] Watterberg KL, Gerdes JS, Gifford KL, Lin HM (1999). Prophylaxis against early adrenal insufficiency to prevent chronic lung disease in premature infants. Pediatrics.

[56] Yeh TF, Chen CM, Wu SY, Husan Z, Li TC, Hsieh WS, Tsai CH, Lin HC (2016). Intratracheal Administration of Budesonide/Surfactant to Prevent Bronchopulmonary Dysplasia. Am J Respir Crit Care Med.

[57] Efird MM, Heerens AT, Gordon PV, Bose CL, Young DA (2005). A randomized-controlled trial of prophylactic hydrocortisone supplementation for the prevention of hypotension in extremely low birth weight infants. J Perinatol.

[58] Vermont Oxford Network Steroid Study Group. (2001). Early postnatal dexamethasone therapy for the prevention of chronic lung disease. Pediatrics.

[59] Sinkin RA, Dweck HS, Horgan MJ, Gallaher KJ, Cox C, Maniscalco WM, Chess PR, D'Angio CT, Guillet R, Kendig JW, Ryan RM, Phelps DL (2000). Early dexamethasone-attempting to prevent chronic lung disease. Pediatrics.

[60] Ng PC, Lee CH, Bnur FL, Chan IH, Lee AW, Wong E, Chan HB, Lam CW, Lee BS, Fok TF (2006). A double-blind, randomized, controlled study of a “stress dose” of hydrocortisone for rescue treatment of refractory hypotension in preterm infants. Pediatrics.

[61] Shah VS, Ohlsson A, Halliday HL, Dunn M (2012). Early administration of inhaled corticosteroids for preventing chronic lung disease in ventilated very low birth weight preterm neonates. Cochrane Database Syst Rev.

[62] Queen MA, Myers AL, Hall M, Shah SS, Williams DJ, Auger KA, Jerardi KE, Statile AM, Tieder JS (2014). Comparative effectiveness of empiric antibiotics for community-acquired pneumonia. Pediatrics.

