# Supplementary material for: Efficacy of glucocorticoids, vitamin A and caffeine therapies for neonatal mortality in preterm infants: a network meta-analysis
Source: Oncotarget. 2017 Sep 14;8(46):81167–75. doi: 10.18632/oncotarget.20882 (PMC5655271; doi:10.18632/oncotarget.20882)
Supplement: Supplementary file 1 [file oncotarget-08-81167-s001.pdf]

Efficacy of glucocorticoids, vitamin A and caffeine therapies for neonatal mortality in preterm infants: a network meta-analysis

SUPPLEMENTARY MATERIALS

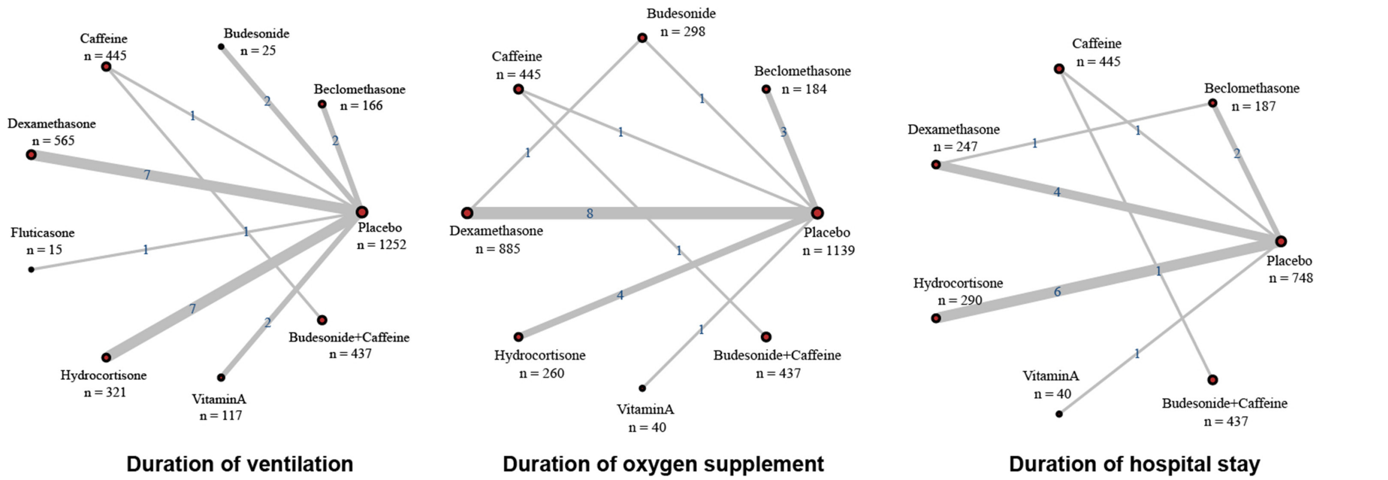

Supplementary Figure 1: Network diagram of duration of ventilation, duration of oxygen supplementation, and duration of hospital stay. Each node represents a therapy; the number beside the nodes represents the number of people involved and the number between two nodes represents the number of study involved in the head-to-head comparison.

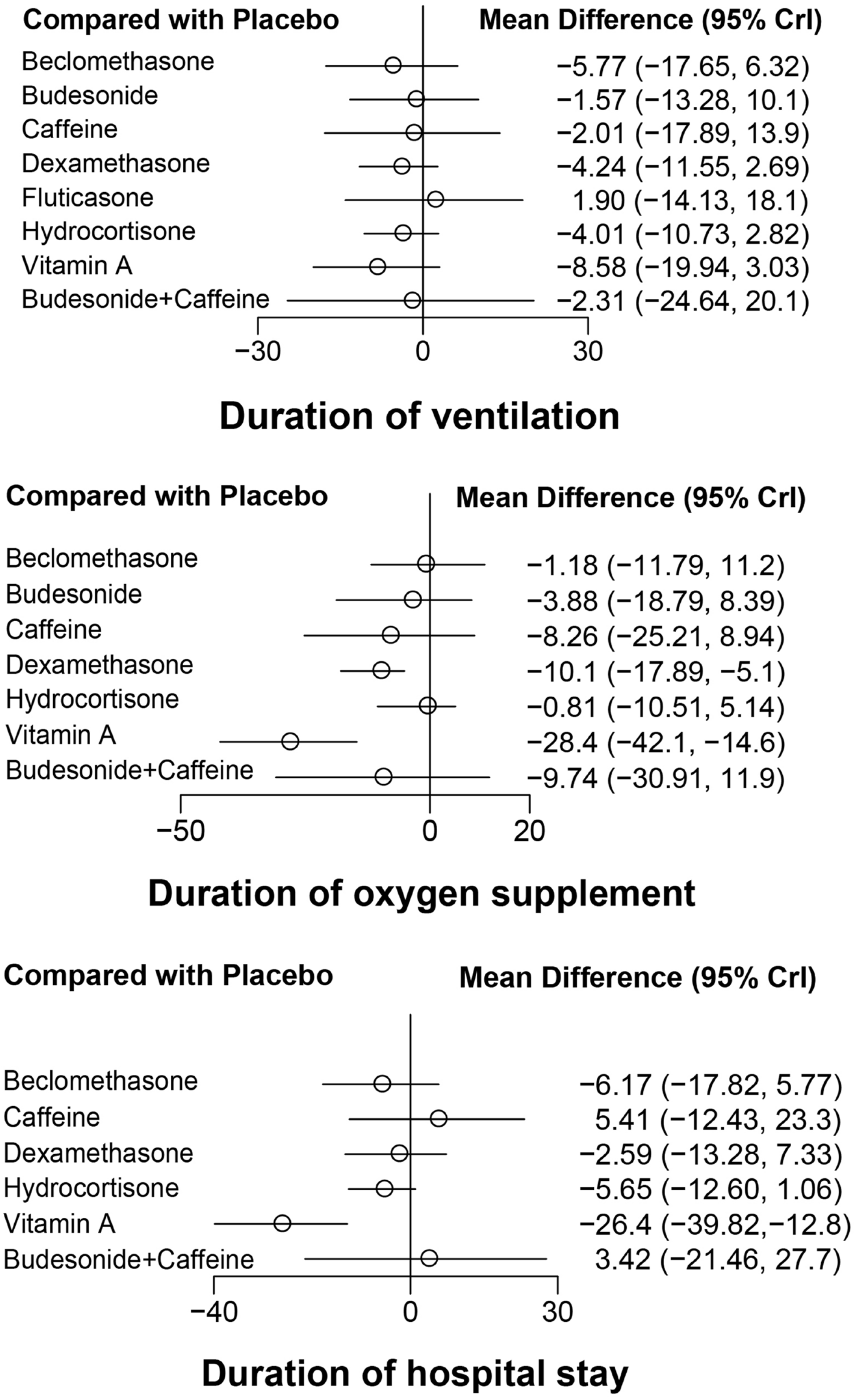

Supplementary Figure 2: Forest plots of duration of ventilation, duration of oxygen supplementation, and duration of hospital stay. Odds ratio (ORs) or mean difference (MD) with corresponding 95% credible intervals (95% CrIs) was used to measure the relative efficacy of different treatments. The existence of significant statistical difference.

Supplementary Table 1: Characteristics of included studies. See Supplementary_Table_1

Supplementary Table 2: Jadad scale for 42 included studies

Author	Year	Blinding	Randomization	Withdrawal	High Quality*
Kugelman	2017	2	2	0	√
Yeh	2016	1	2	0	√
Nakamura	2016	2	2	1	√
Baud	2016	2	2	1	√
Armanian	2016	2	2	1	√
Bassler	2015	0	2	1	√
Kiatchoosakun	2014	1	1	1	√
Parikh	2013	1	1	1	√
Benn	2010	2	2	0	√
Bonsante	2007	2	2	1	√
Schmidt	2006	1	2	1	√
Ng	2006	2	2	0	√
Doyle	2006	1	2	1	√
Peltoniemi	2005	2	2	1	√
Efird	2005	2	2	1	√
Anttila	2005	2	2	1	√
Watterberg	2004	2	2	1	√
Walther	2003	2	2	0	√
Rozycki	2003	2	2	1	√
Ravishankar	2003	2	2	1	√
Jangaard	2002	2	2	1	√
Beresford	2002	2	1	1	√
Wardle	2001	2	2	1	√
Stark	2001	2	2	1	√
Halliday	2001	1	2	1	√
VONSSG	2001	2	2	1	√
Sinkin	2000	2	2	1	√
Jonsson	2000	2	2	1	√
Watterberg	1999	2	2	0	√
Tyson	1999	1	2	1	√
Romagnoli	1999	1	2	1	√
Merz	1999	2	2	1	√
Lin	1999	2	2	1	√
Kothadia	1999	2	2	1	√
Garland	1999	2	2	1	√
Fok	1999	1	2	1	√
Tapia	1998	2	1	0	√
Denjean	1998	2	2	0	√
Subhedar	1997	1	2	1	√
Rastogi	1996	2	2	0	√
Brozanski	1995	2	2	1	√

*When total score ≥ 3, the relative RCT will be considered as of high quality. The questions were as follows: 1. Was the study described as randomized? 2. Was the study described as double blind? 3. Was there a description of withdrawals and dropouts? To receive the corresponding point, an article should describe the number of withdrawals and dropouts, in each of the study groups, and the underlying reasons. Additional points were given if: 1. The method of randomization was described in the paper, and that method was appropriate. (1 extra point in randomization part); 2. The method of blinding was described, and it was appropriate. (1 extra point in blinding part).
